# Creation and Validation of the Japanese Cute Infant Face (JCIF) Dataset

**DOI:** 10.3389/fpsyg.2022.819428

**Published:** 2022-02-18

**Authors:** Hiroshi Nittono, Akane Ohashi, Masashi Komori

**Affiliations:** ^1^Graduate School of Human Sciences, Osaka University, Suita, Japan; ^2^Faculty of Information and Communication Engineering, Osaka Electro-Communication University, Neyagawa, Japan

**Keywords:** cuteness, kawaii, infant face, perceptual discrimination, sex, age

## Abstract

Research interest in cuteness perception and its effects on subsequent behavior and physiological responses has recently been increasing. The purpose of the present study was to produce a dataset of Japanese infant faces that are free of portrait rights and can be used for cuteness research. A total of 80 original facial images of 6-month-old infants were collected from their parents. The cuteness level of each picture was rated on a 7-point scale by 200 Japanese people (100 men and 100 women in their 20s–60s). Prototypical high- and low-cuteness faces were created by averaging the top 10 and bottom 10 faces according to the mean cuteness ratings. Then, 50 composite faces were made by mixing two faces randomly chosen from the 60 unused middle-cuteness faces. The normative cuteness ratings of these composite faces were obtained from 229 Japanese men and women in their 20s–60s. The shape of each composite face was transformed to be cuter (+50%) or less cute (–50%) along a continuum between the high- and low-cuteness prototypical faces. A two-alternative forced-choice task (*N* = 587) confirmed that cuteness discrimination was better than the chance level for all 50 face pairs. Moreover, the results showed that young men had poorer sensitivity to cuteness differences in infant faces than older men and women of any age. This Japanese Cute Infant Face (JCIF, “jay-sif”) dataset, including composite face images and normative rating scores, is publicly available online.

## Introduction

Cuteness is an emerging field of research and has been attracting attention in various areas, such as psychology (Sherman and Haidt, 2011; Nittono, 2016), neuroscience (Kringelbach et al., 2016), humanities (Dale et al., 2017; May, 2019), marketing (Nenkov and Scott, 2014), and engineering (Marcus et al., 2017). The objects of cuteness include babies and animals (Little, 2012; Borgi and Cirulli, 2016), industrial designs (Miesler et al., 2011), robots (Caudwell et al., 2019), and foods (Lee et al., 2018). In English, cuteness is regarded as “infant physical attractiveness” (Karraker and Stern, 1990), which is a trait of an object and something to be perceived. Scientific research on cuteness and the response to it can be traced back to Lorenz (1943) conception of *Kindchenschema*. He proposed that certain physical elements induce specific actions in animals. For a human example of these innate releasing mechanisms, he noted that several physical features evoke affective responses related to caregiving. According to his introspection, these include a round face, high and protruding forehead, soft body surface, and so forth. The class containing these elements can be called “cuteness,” while each element within the class can be called a “cuteness cue.” Cognitive and affective responses to specific features typical of infant faces are assumed to have a biological and evolutionary foundation (Kringelbach et al., 2016).

Research shows that the level of cuteness in infant faces is associated with a large forehead, small chin, full lips, and chubby round features (Hildebrandt and Fitzgerald, 1979b; Almanza-Sepúlveda et al., 2018). Conversely, cuteness levels can be experimentally manipulated by changing these shapes, and two types of manipulations have been used. The first method is to edit each element in a face directly, such as by creating a rounder face, higher forehead, bigger eyes, or smaller nose and mouth (Sternglanz et al., 1977; Alley, 1981; Glocker et al., 2009a; Borgi et al., 2014; Bos et al., 2018; Endendijk et al., 2018; Löwenbrück and Hess, 2021). The other method is to transform a face shape on a cuteness dimension by connecting the physical characteristics of the prototypical high- and low-cuteness faces (Sprengelmeyer et al., 2009; Lobmaier et al., 2010; Hahn et al., 2013). Each prototypical face is obtained by averaging several faces that have been rated with the highest or lowest cuteness levels by a group of people. A face becomes cuter when transforming it to be closer to the high-cuteness face and less cute when transforming it to be closer to the low-cuteness face. Both methods are similar in that they change physical features related to the perception of cuteness, and both methods have been shown to produce expected effects.

Although the former method is implemented more easily because it does not require a presurvey for making prototypical faces, arbitrariness exists in selecting the elements to be edited. In contrast, the latter method can produce more natural images because the transformation is done within empirically defined cuteness dimensions. In studies, when compared to infant faces that have been manipulated to be less cute, infant faces that have been manipulated to be cuter receive higher cuteness ratings (Glocker et al., 2009a; Borgi et al., 2014), are associated with greater efforts to see them longer (Hahn et al., 2013), activate reward-related brain regions (Glocker et al., 2009b; but see Bos et al., 2018; Endendijk et al., 2020 for null results), and modulate the amplitude of brain electrical responses reflecting early visual processing (N170 and P200, occurring less than 250 ms; Hahn et al., 2016; but see Endendijk et al., 2018, for a null result).

The perceptual ability to detect differences in cuteness levels varies across individuals and situations (Hahn and Perrett, 2014; DeBruine et al., 2016). There is some evidence that sex and age influence discrimination sensitivity to cuteness in infant faces (e.g., Sprengelmeyer et al., 2009; Lobmaier et al., 2010). For example, Sprengelmeyer et al. (2009) reported that younger women were able to detect the difference between cuter and less cute infant faces more accurately than were men and older women. Moreover, they suggested that female reproductive hormones, such as progestogen and estrogen, contribute to cuteness sensitivity, based on their findings that women showed less sensitivity after menopause, even when their age was matched, and that women who took contraceptives that artificially increase levels of progestogen and estrogen showed better discrimination than those who did not. Menstrual phases have also been shown to affect sensitivity, which was higher in the follicular phase than in the luteal phase (Lobmaier et al., 2015; but see Sprengelmeyer et al., 2013, for a null result). However, no evidence of an association between cuteness sensitivity and salivary testosterone, estradiol, and progesterone levels has been obtained to date (Hahn et al., 2015a; Lobmaier et al., 2015).

The present study had two purposes. First, we developed a new stimulus dataset of infant faces that can be used in future cuteness research—the Japanese Cute Infant Face (JCIF, “jay-sif”) dataset. It consists of 50 composite faces and three average faces of 6-month-old infants (called “base” faces), along with manipulated versions that augmented or reduced features related to cuteness perception according to prototypical high-cuteness and low-cuteness faces. The age of 6 months was selected based on the finding that the perceived cuteness of infant faces peaked around 6 months old (Franklin and Volk, 2018; Franklin et al., 2018). To make the facial images freely available, composite faces without portrait rights were created. Original facial images of 6-month-old infants were collected in Japan, and their cuteness levels were rated by Japanese people. According to the mean scores, faces rated to be higher or lower in cuteness were selected and averaged to create the prototypical high- and low-cuteness faces. These images were used as reference points for face shape manipulation to create versions that are cuter (i.e., transformed the face closer to the high-cuteness face shape, +50%) or less cute (i.e., transformed the face closer to the low-cuteness face shape, –50%). Normative cuteness ratings of the base faces and the accuracy of discriminating cuteness level between the high- and low-cuteness versions of the faces were obtained from a Japanese population.

Second, the data obtained on cuteness rating and discrimination accuracy were scrutinized in terms of the effects of sex and age on an individual’s ability for cuteness perception. The data were separately analyzed for 10 subgroups of men and women in their 20s, 30s, 40s, 50s, and 60s. According to Sprengelmeyer et al. (2009), a significant interaction between sex and age would be expected, with a larger difference by sex (i.e., women are more accurate than men) found in younger age groups. Additionally, the effect of parental status was examined to see if having one or more children was associated with a better perceptual ability for cuteness.

## Materials and Methods

### Outline

This study consisted of the following six steps: (1) collecting original infant face images, (2) obtaining cuteness ratings of the original faces, (3) creating composite faces using image processing, (4) obtaining normative cuteness ratings, (5) conducting a discrimination task, and (6) analyzing the data in terms of sex, age, and parental status. The procedure for each step is described in detail in the following sections. The research protocol was approved by the Behavioral Research Ethics Committee of the Osaka University School of Human Sciences (HB30-033). Electronic informed consent was obtained from all participants via online surveys.

### Collecting Original Infant Face Images

Colored pictures of real infant faces (6 ± 1 months old) were collected from their parents with informed consent. Recruiting was based on the first author’s personal connection and the snowball sampling method. The entry conditions were photos in which the infant was front-facing, mouth-closed, with a neutral expression, and the full outline of the face was visible without any accessories. The submission of a picture was rewarded by a cash voucher of 500 Japanese yen. Initially, 89 images were collected. Among these, 40 male and 40 female infants were selected according to an image quality evaluation by four persons, including the first and second authors. Although this study did not deal with the sex of infant models because it is often misclassified (Hildebrandt and Fitzgerald, 1979a), the same number of male and female infants’ faces were used to avoid any potential bias. Only the face area was clipped after correcting the tilt by rotating the face to make the line connecting the pupils horizontal. The image size was adjusted to 1,024 × 1,024 pixels. For each face, 179 landmark points were manually determined using Psychomorph software (Tiddeman et al., 2001; Sutherland, 2015).^1^ For online surveys, the image size was reduced to 256 × 256 pixels to keep the file size small enough to ensure smooth loading and display on various devices.

### Obtaining Cuteness Ratings of the Original Faces

The levels of cuteness of these 80 face images were rated on a 7-point scale, where 1 = *Not cute* (kawaii) *at all* and 7 = *Extremely cute* (kawaii), using Qualtrics (Seattle, WA, United States), a crowd-based questionnaire software platform. The facial images were presented one-by-one in a random order. A total of 200 respondents (20 men and 20 women in each of their 20s, 30s, 40s, 50s, and 60s) were recruited by Cross Marketing Group, Inc. (Tokyo, Japan) for an honorarium stipulated by the company. The online data collection was conducted November 14–15, 2018.

Before closing the presurvey, a total of 256 respondents answered. First, 18 of the responses were removed because they gave the same rating score to all faces. Among the remaining 236 respondents, 36 were removed so that each sex and age subgroup consisted of the same number of 20 respondents to avoid unbalanced contributions of a particular sex or age. This was done by retaining those who took longer to complete the questionnaire (i.e., assumed to be more prudent). The cuteness score of each face was calculated by averaging the ratings of all respondents. The cuteness scores calculated from the final 200 respondents were almost identical and highly correlated with those calculated from the untrimmed 236 respondents (*r* = 0.99).

### Creating Composite Faces Using Image Processing

According to the mean cuteness scores, the 10 cutest and 10 least cute faces were selected. Incidentally, the 10 most and 10 least cute faces consisted of 5 male and 5 female babies, respectively. Prototypical high- and low-cuteness faces were created by averaging them using Psychomorph software. Then, 50 composite faces (25 male and 25 female) were made by averaging two randomly selected same-sex faces from the middle-ranked 30 male and 30 female babies who were not used for creating prototype faces. The face, including the ears and hair, was clipped on a black background. The image quality of the composite faces was checked by nine independent raters. Composite faces that were rated as unnatural by more than two raters were discarded and replaced by other faces. In addition, images were created for the average of the 60 middle-ranked faces (A60), the average of the 30 middle-ranked female babies’ faces (F30), and the average of the 30 middle-ranked male babies’ faces (M30). Then, the color, tone, and average brightness of these face images were adjusted to the mean of the 80 original images. Landmark positions were manually corrected to fit the outline of the composite face image.

The shape of these 53 created faces (i.e., 50 composite and 3 average faces; called the base or 0% faces) were transformed to a cuter (+50%) or less cute (–50%) version using the two prototype faces using Psychomorph software. Color and texture information were not used for the transformation. In this study, cuter faces were operationally defined as faces closer to the prototypical high-cuteness face, and less cute faces were defined as faces closer to the prototypical low-cuteness face.

### Obtaining Normative Cuteness Ratings

A total of 260 Japanese people between 20 and 69 years old were recruited by Cross Marketing Group, Inc. (Tokyo, Japan) and received an honorarium stipulated by the company. The survey was conducted online using Qualtrics from December 6, 2018 to December 10, 2018. The participants rated a total of 61 faces in two blocks. First, each of 50 composite faces was presented in a random order, and respondents were asked to rate each face’s cuteness on the same 7-point scale used in the presurvey. Next, participants rated the 11 average faces presented one-by-one in a random order: three average base faces (i.e., A60, F30, and M30), their high- and low-cuteness versions (i.e., A60+50, A60–50, F30+50, F30–50, M30+50, and M30–50), and prototypical high- and low-cuteness faces. Out of the 260 responses, 229 were retained for analysis after excluding those who met any of the following conditions: (1) rated all imaged with the same number, (2) took too short a time (< 2 min) to answer the entire questionnaire, (3) answered that they had a grandchild despite stating that they were in their 20s, or (4) answered that they had a grandchild despite stating that they had no children. Table 1 shows the constitution of the sample.

**TABLE 1:** Summary of survey samples.

	Presurvey			Rating			Discrimination		
Age group	Female	Male	Total	Female	Male	Total	Female	Male	Total
20s	20	20	40	22	21	43	60	52	112
30s	20	20	40	23	26	49	59	58	117
40s	20	20	40	20	24	44	60	61	121
50s	20	20	40	23	22	45	59	57	116
60s	20	20	40	26	22	48	58	63	121
Sum	100	100	200	114	115	229	296	291	587

### Conducting a Discrimination Task

A total of 661 Japanese people aged between 20 and 69 years who had not participated in the normative rating survey were recruited by the same company and paid for their participation. To obtain reliable estimates, more than 50 participants were collected in each of the 10 Sex × Age subgroups (i.e., men and women in their 20s, 30s, 40s, 50s, and 60s). The online data collection was conducted December 6–7, 2018. The participants conducted a total of 65 trials via three tasks. In the first task, 50 pairs of +50% and –50% versions of the 50 composite faces were presented in a random order. Respondents were asked to choose the cuter (more kawaii). In the second task, +50%, 0% (base), and –50% versions of each average face (F30, M30, and A60) were compared in a round-robin system (i.e., +50% vs. 0%, 0% vs. –50%, and +50% vs. –50%). In addition, the prototypical high- and low-cuteness faces were compared directly. Therefore, 10 comparisons were made in total in random order. Respondents were asked to choose the cuter (more kawaii). The third task was conducted to confirm whether the respondent watched the stimulus images and answered conscientiously. Five pairs of adult male faces and infant faces, taken from different sources, were presented (see Supplementary Figure 1 for an example), and the respondents were asked to select the infant face. Among the initial 611 respondents, 587 were retained after excluding those who met any of the following conditions: (1) made at least one mistake in the third task (confirmation test), (2) took too short a time (< 2 min) to answer the entire questionnaire, (3) answered that they had a grandchild despite stating that they were in their 20s, or (4) answered that they had a grandchild despite having stated that they had no children. Table 1 shows the constitution of the sample.

### Analyzing the Data in Terms of Sex, Age, and Parental Status

Cuteness rating scores were subjected to an analysis of variance (ANOVA) with factors of sex (female and male) and age (20s, 30s, 40s, 50s, and 60s). Dependent variables were the mean rating of the 80 original infant faces and the mean rating of the 50 composite faces. Moreover, two additional ANOVAs were conducted for the main survey. First, the cuteness ratings of the prototypical high- and low-cuteness faces were compared using a Prototypical Cuteness (high and low) × Sex × Age ANOVA. Second, the effect of shape transformation on perceived cuteness was examined using a Manipulation Level (+50%, 0%, and –50%) × Sex × Age ANOVA.

Discrimination accuracy was similarly subjected to a Sex × Age ANOVA. For the individual composite faces, the accuracy of choosing the cuter one from the two faces was calculated as a percentage (out of 50 pairs) for each respondent. For the prototypical face pair, detection accuracy was a binary value (0 = wrong, 1 = correct). For manipulated faces, three types of average faces (A60, F30, and M30) were pooled. Then, accuracy was analyzed using a Pair (+50% vs. 0%, 0% vs. –50%, and +50% vs. –50%) × Sex × Age ANOVA. Moreover, the scale value (in *z* score) of each manipulated face was calculated according to Thurstone’s paired comparison procedure (Case V) for each sex and age group (Thurstone, 1927). The above protocol was registered before conducting the analysis at https://osf.io/pseym/. There were two deviations from the preregistered protocol. First, the term “sex” rather than “gender” is used throughout this paper. Although gender as a social role may affect it, cuteness perception has a biological and evolutionary background, as described in the Introduction. Second, manipulated face pairs were not pooled by the level of difficulty (e.g., 100% difference pair and 50% difference pair). Although previous studies pooled the data of the +50% vs. 0% and 0% vs. –50% pairs as the 50% difference pairs (Sprengelmeyer et al., 2009; Lobmaier et al., 2010, 2015), it was reasonable to examine whether any systematic difference in discrimination accuracy was observed between the +50% vs. 0% pairs and the 0% vs. –50% pairs. Whenever the degree of freedom of a within-participants factor was more than two, the Greenhouse-Geisser ε correction for the violation of sphericity assumption was applied. *Post hoc* comparison was made by paired *t*-tests with Bonferroni correction.

To examine the effect of parental status on cuteness discrimination sensitivity, a hierarchical multiple linear regression analysis was conducted because parental status interacts with age. In this analysis, numerical age, rather than age group, was used as a continuous variable. The mean accuracy of the 50 composite faces was used as the response variable. As the first step, sex, age, and their interaction term were entered. Then, as a second step, parental status (0 = no child, 1 = one or more children) was entered to determine if there would be a significant increase in explained variance. Finally, all the related interaction terms (Parental Status × Sex, Parental Status × Age, and Parental Status × Sex × Age) were entered in a stepwise fashion. To avoid the risk of multicollinearity, all variables creating interaction terms were centered at their means. All statistical analyses were conducted with SPSS Statistics 24 software (IBM Corp., Armonk, NY, United States). The significance level was set at 0.05.

## Results

### Presurvey

**FIGURE 1:**
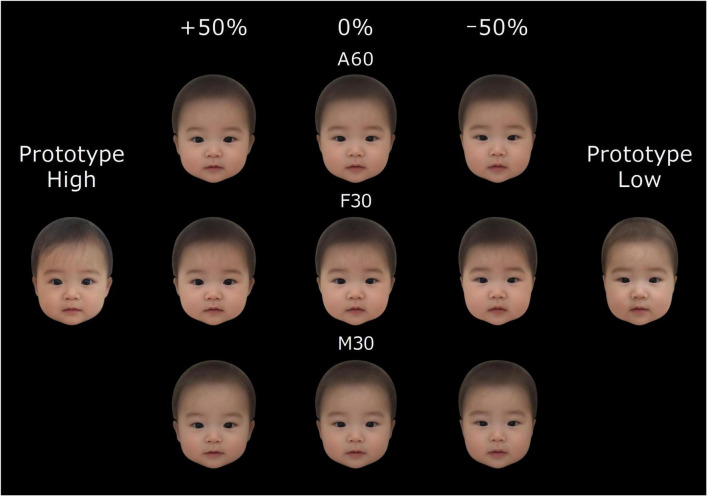
Average faces (0%), manipulated faces (+50%: cuter, –50%: less cute), and prototypical high- and low-cuteness faces. A60: Average face of 30 female and 30 male babies. F30: Average face of 30 female babies. M30: Average face of 30 male babies. The facial images are computer generated.

The mean cuteness ratings of the original faces (*N* = 200) ranged from 3.09 to 4.73 on a 7-point scale (*M* = 3.74, *SD* = 0.39). Supplementary Figure 2 shows the mean and 95% confidence interval (CI) of each of the 80 original faces. The mean ratings of the 10 cutest and 10 least cute faces were 4.39 (*SD* = 0.15, range = 4.18–4.73) and 3.15 (*SD* = 0.05, range = 3.09–3.21), respectively. The former were lower and the latter were higher than the midpoint (4). Figure 1 shows the prototypical high- and low-cuteness faces created by averaging these sets of faces.

### Japanese Cute Infant Face Dataset

The dataset includes 50 composite faces, three types of average faces (A60, F30, and M30), and their manipulated faces that were transformed into a cuter (+50%) or less cute (--50%) version. All of the face images and templates with 179 landmarks are publicly available.^2^ Figure 1 shows three types of average faces and their shape-transformed versions.

**FIGURE 2:**
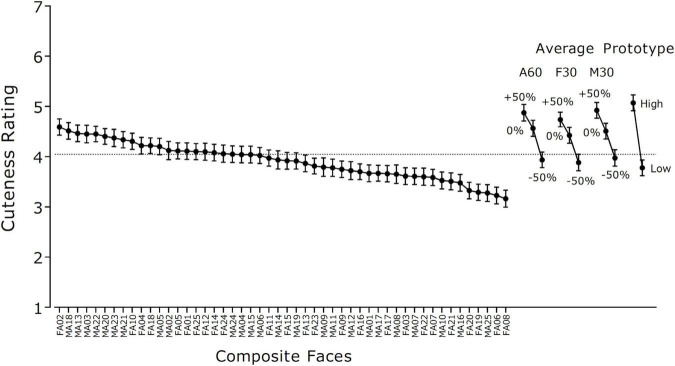
Mean cuteness rating scores for 50 composite faces, average faces (0%), manipulated faces (+50%, –50%), and prototypical high- and low-cuteness faces (*N* = 229). 1 = *not cute* (kawaii) *at all*, 7 = *extremely cute* (kawaii). Error bars indicate 95% confidence intervals. A60: Average face of 30 female and 30 male babies. F30: Average face of 30 female babies. M30: Average face of 30 male babies.

Figure 2 shows the mean cuteness ratings and their 95% CIs (*N* = 229) of 50 composite faces, three types of average faces (0%), their manipulated versions (+50% and –50%), and two prototype faces. The cuteness scores of the composite faces ranged from 3.16 to 4.59 (*M* = 3.91, *SD* = 0.37). Although this mean was slightly higher than the mean of the 80 original faces, *t*(128) = 2.49, *p* = 0.014, the variance of the scores did not differ significantly between the presurvey and the main survey, *F*(79, 49) = 1.13, *p* = 0.325, suggesting that the composite faces were as varying as the original faces in terms of cuteness level. Average and prototype faces were generally rated as cuter than individual composite faces. A detailed analysis of the effect of face shape manipulation on cuteness ratings will be reported later.

**FIGURE 3:**
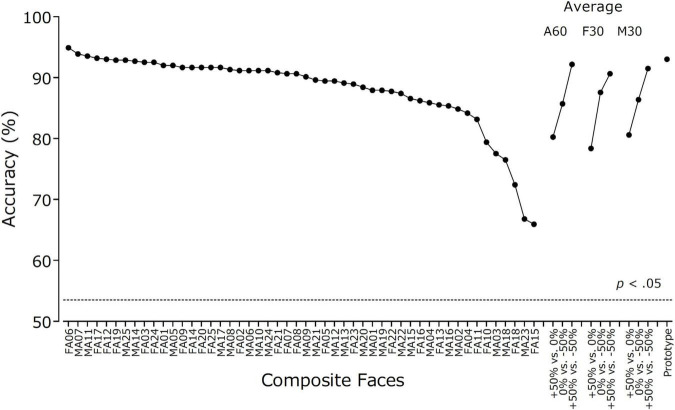
Discrimination accuracy for manipulated high- vs. low-cuteness versions of 50 composite faces and three types of average faces (*N* = 587). Accuracy for prototypical high- vs. low-cuteness faces is also shown. The dotted line shows the threshold of statistical significance computed from the binomial distribution.

Figure 3 shows the accuracy of discriminating between cuter (+50%) and less cute (–50%) versions of the faces (*N* = 587). The mean accuracy for 50 composite faces ranged from 65.9 to 94.9% (*M* = 88.0%, *SD* = 6.4). All the face pairs could be successfully discriminated better than chance (critical levels = 53.5 and 56.6%, one-tailed *p* < 0.05 and *p* < 0.001, respectively, according to the binomial distribution). The correlation between the mean cuteness rating and discrimination accuracy of these composite faces was *r* = –0.42 (*p* = 0.002). The scattergram is shown in Supplementary Figure 3. The results showed that base faces with lower cuteness ratings were associated with higher discrimination accuracies. That is, shape manipulation was more effective for less cute faces than for cuter faces.

### Effects of Sex and Age on Cuteness Ratings

**FIGURE 4:**
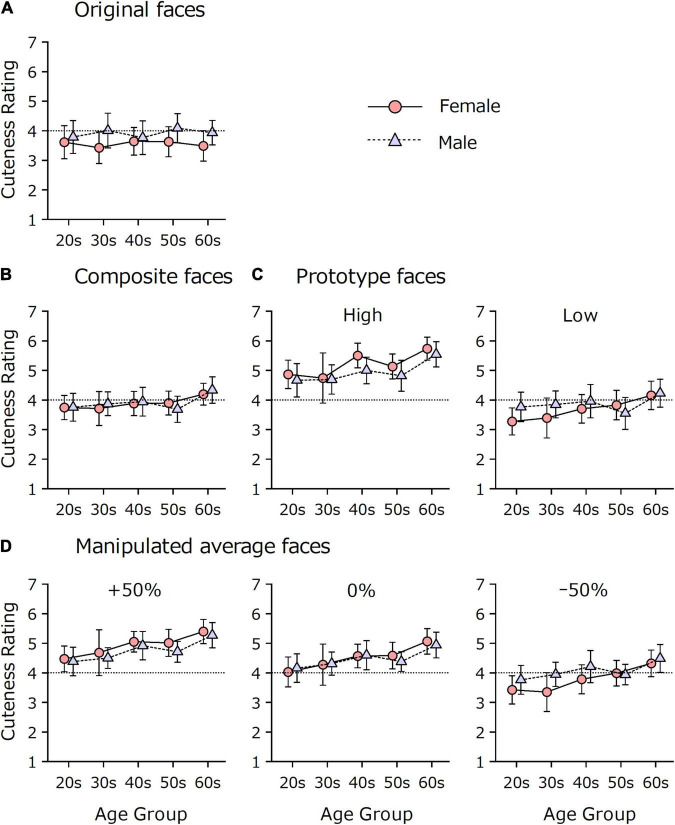
Sex and age differences in cuteness ratings. 1 = *not cute* (kawaii) *at all*, 7 = *extremely cute* (kawaii). Error bars indicate 95% confidence intervals. **(A)** The data on 80 original faces. **(B)** The data on 50 composite faces. **(C)** The data on prototype faces. **(D)** The data on manipulated average faces.

Figure 4A shows the effects of sex and age on the mean cuteness rating of the original 80 faces. A Sex × Age ANOVA revealed a significant main effects of sex, *F*(1, 190) = 5.09, *p* = 0.025, η*_*p*_*^2^ = 0.026. Women gave lower ratings (*M* = 3.56, *SD* = 1.09, 95% CI [3.34, 3.77]) than men (*M* = 3.91, *SD* = 1.11, 95% CI [3.69, 4.13]). The main effect of age and the Sex × Age interaction effect were not significant, *F*s < 1.

Figure 4B shows the mean cuteness rating of the 50 composite faces. All the 95% CI intervals crossed the midpoint, indicating that both men’s and women’s mean ratings did not differ from the midpoint. No significant main effect of sex was obtained, *F* < 1. The main effect of age and the interaction effect were also not significant *F*(4, 219) = 2.02, *p* = 0.093, η*_*p*_*^2^ = 0.036; *F* < 1, respectively.

**FIGURE 5:**
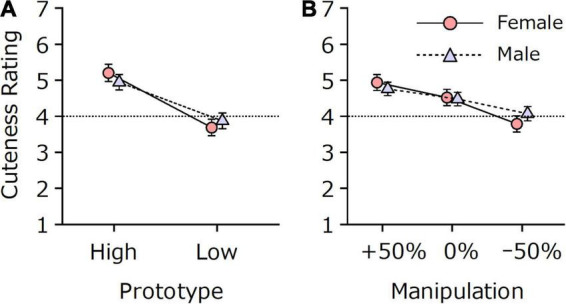
Sex differences in cuteness ratings of prototype and manipulated faces. 1 = *not cute* (kawaii) *at all*, 7 = *extremely cute* (kawaii). Error bars indicate 95% confidence intervals. **(A)** The data on prototype faces. **(B)** The data on manipulated average faces.

Figure 4C shows that the prototypical high-cuteness face was rated as cuter than the prototypical low-cuteness face, as expected. A Prototypical Cuteness × Sex × Age ANOVA revealed a significant effect of prototypical cuteness, *F*(1, 219) = 239.19, *p* < 0.001, η*_*p*_*^2^ = 0.522, confirming that the high-cuteness face was rated to be cuter than the low-cuteness face. Moreover, the effect of age was significant, *F*(4, 219) = 4.68, *p* = 0.001, η*_*p*_*^2^ = 0.079, suggesting that older groups tended to give higher cuteness ratings. The main effect of sex and the interaction effects, except for the Prototypical Cuteness × Sex interaction, were not significant, *F*s < 1. Figure 5A illustrates the Prototypical Cuteness × Sex interaction, *F*(1, 219) = 7.08, *p* = 0.008, η*_*p*_*^2^ = 0.031. The difference between high- and low-cuteness faces was greater for women (*M* = 1.52, *SD* = 1.26, 95% CI [1.28, 1.75]) than for men (*M* = 1.07, *SD* = 1.25, 95% CI [0.84, 1.30]).

Figure 4D shows the effects of sex and age on cuteness ratings separately by the level of manipulation. The augmented faces (+50%) were rated to be cuter than the reduced faces (–50%), and the base faces (0%) were positioned in between. A Manipulation Level × Sex × Age ANOVA showed significant main effects of manipulation level and age, *F*(2, 438) = 198.68, *p* < 0.001, ε = 0.72, η*_*p*_*^2^ = 0.48; *F*(4, 219) = 5.12, *p* = 0.001, η*_*p*_*^2^ = 0.09, respectively. The main effect of sex and the interaction effects, except for the Manipulation Level × Sex interaction, were not significant, *F*s < 1. Figure 5B illustrates the Manipulation Level × Sex interaction, *F*(2, 438) = 12.99, *p* < 0.001, ε = 0.72, η*_*p*_*^2^ = 0.06. As with the prototype faces, women showed a greater effect from face manipulation than men. The difference between +50% and –50% was larger for women (*M* = 1.15, *SD* = 0.99, 95% CI [0.96, 1.33]) than for men (*M* = 0.68, *SD* = 0.76, 95% CI [0.54, 0.82]). When the difference between +50% and 0% faces and the difference between 0% and –50% faces were compared with a Pair (+50% and 0%, 0% and –50%) × Sex × Age ANOVA, the main effect of sex was significant, *F*(1, 219) = 15.47, *p* < 0.001, η*_*p*_*^2^ = 0.07, suggesting that women distinguished faces with different cuteness levels more finely than men. Moreover, the main effect of pair was significant, *F*(1, 219) = 18.31, *p* < 0.001, η*_*p*_*^2^ = 0.08. The score difference was larger for the 0% and –50% pair (*M* = 0.57, *SD* = 0.66, 95% CI [0.48, 0.65]) than for the +50% and 0% pair (*M* = 0.35, *SD* = 0.52, 95% CI [0.28, 0.41]). These results suggest that even when the size of the physical difference was identical, the manipulation to reduce cuteness had a greater effect on ratings than the manipulation to augment cuteness. The main effect of age and the other interaction effects were not significant (*p*s > 0.074).

### Effects of Sex and Age on Cuteness Discrimination

**FIGURE 6:**
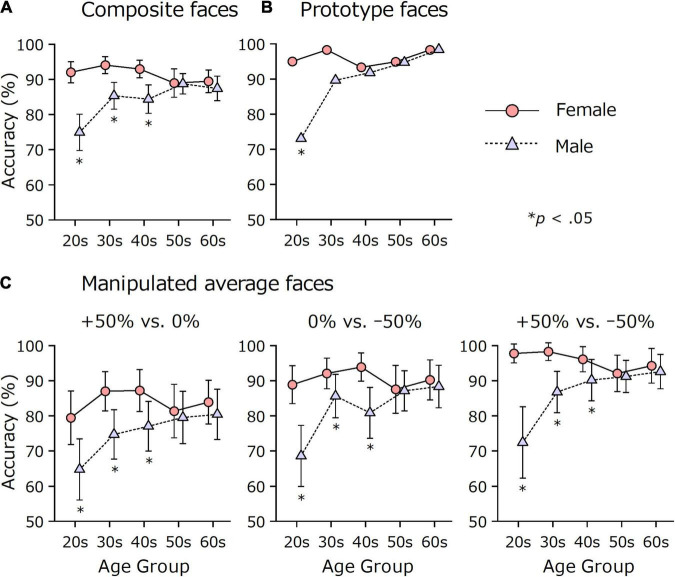
Sex and age differences in cuteness discrimination accuracy. Participants (*N* = 587) were asked to choose the cuter face from the pair. Error bars indicate 95% confidence intervals. Note that the accuracy for prototype faces has no error bar because the value indicates the proportion of respondents who answered correctly on a single trial. **(A)** The data on 50 composite faces. **(B)** The data on prototype faces. **(C)** The data on manipulated average faces.

Figure 6A shows the effects of sex and age on the accuracy of discriminating between the +50% and –50% versions of 50 composite faces. Young men showed lower accuracy than women and older men. A Sex × Age ANOVA showed significant main effects of sex and age and their interaction effect, *F*(1, 577) = 43.61, *p* < 0.001, η*_*p*_*^2^ = 0.07; *F*(4, 577) = 3.82, *p* = 0.004, η*_*p*_*^2^ = 0.03; *F*(4, 577) = 7.04, *p* < 0.001, η*_*p*_*^2^ = 0.05, respectively. When analyzed separately, men showed a significant age effect, *F*(4, 286) = 7.24, *p* < 0.001, η*_*p*_*^2^ = 0.09, while women did not, *F*(4, 291) = 2.02, *p* = 0.092, η*_*p*_*^2^ = 0.03). Sex differences were significant in the 20s, 30s, and 40s (*p*s < 0.001), but not in the 50s and 60s (*p*s > 0.392). The largest difference was found in the 20s. Women answered correctly (*M* = 92.0%, *SD* = 11.7, 95% CI [89.0, 95.0]) more than men (*M* = 74.9%, *SD* = 18.6, 95% CI [69.7, 80.1]), and the effect size was large (*d* = 1.12).

A similar trend in which young men were less sensitive to cuteness differences was found in other stimulus sets. For the comparison of the prototype faces (Figure 6B, only one trial for each participant), young men showed lower correct rates. The number of respondents who answered correctly was 57 out of 60 women and 38 out of 52 men in their 20s (*p* = 0.001) and 58 out of 59 women and 52 out of 58 men in their 30s (*p* = 0.061), according to Fisher’s exact test.

Likewise, the data on average faces (Figure 6C) showed a similar result. A Pair × Sex × Age ANOVA showed significant main effects of sex and age and their interaction effect, *F*(1, 577) = 33.45, *p* < 0.001, η*_*p*_*^2^ = 0.06; *F*(4, 577) = 5.47, *p* < 0.001, η*_*p*_*^2^ = 0.04; *F*(4, 577) = 5.05, *p* = 0.001, η_*p*_^2^ = 0.03, respectively, which resembled the results of the ANOVA for the 50 composite faces. The main effect of pair was also significant, *F*(2, 1154) = 57.44, *p* < 0.001, ε = 0.96, η*_*p*_*^2^ = 0.09. A *post hoc* comparison showed that all of the pairs differed from each other (*p* < 0.001). The mean accuracies and 95% CIs were 79.5% [77.3, 81.7], 86.3% [84.4, 88.2], and 91.2% [89.5, 92.8] for the +50% vs. 0%, 0% vs. –50%, and +50% vs. –50% pairs, respectively. Even though the size of the physical difference was identical (50%), the +50% vs. 0% pair was more difficult to discriminate than the 0% vs. –50% pair, indicating that the base faces (0%) were perceived as closer to cuter faces (+50%) than to less cute faces (–50%). This finding was supported by the analysis of the scale value (in *z* score) of each manipulated face, calculated according to Thurstone’s paired comparison procedure. On average, the scale values were +1.16, +0.15, and –1.30 for the +50%, 0%, and –50% faces, respectively. Supplementary Figure 4 shows the scale value for each sex and age group. The 0% faces were associated with positive scale values constantly across sex and age, and the scale value difference was larger between –50% and 0% faces than between the 0% and +50% faces. These results indicate that the manipulation to reduce cuteness was more salient than the manipulation to augment cuteness.

### Effects of Parental Status on Cuteness Discrimination

The percentage of respondents with a child was lower in younger generations. The numbers of parent or non-parent respondents are shown in Supplementary Table 1. Table 2 summarizes the results of the hierarchical multiple linear regression. For the first model, sex, age, and their interaction term were entered as predictor variables. As expected from the ANOVA results, the model was statistically significant (*p* < 0.001). For the second model, the predictor of parental status (0 = no, 1 = yes) was added to determine whether this variable improved the prediction. The *R*^2^-value increased significantly, *F*(1, 582) = 4.04, *p* = 0.045. The regression coefficient of parental status was positive (*B* = 2.48, 95% CI [0.06, 4.90]), indicating that having a child was associated with higher discrimination accuracy, although the size of the increase was small (about 2.5%). Then, the interaction terms including parental status were entered in a stepwise fashion. As a result, the predictor of parental status by age (centered at their means) was entered into the third model, with a significant increase in the *R*^2^-value, *F*(1, 581) = 3.88, *p* = 0.049. The regression coefficient of this interaction term was negative (*B* = –0.18, 95% CI [–0.35, –0.00]), indicating that the enhancing effect of parental status on cuteness discrimination accuracy reduced as age increased. Supplementary Figure 5 shows the relationship between parental status and cuteness discrimination accuracy by sex and age group.

**TABLE 2:** Summary of hierarchical linear regression analysis for variables predicting cuteness discrimination accuracy (*N* = 587).

	Model 1				Model 2				Model 3			
Variable	*B*	*B SE*	β	*t*	*B*	*B SE*	β	*t*	*B*	*B SE*	β	*t*
Constant	80.07	2.00			80.68	2.02			81.46	2.06		
Sex	7.24	1.12	0.25	6.47***	6.91	1.13	0.24	6.12***	6.81	1.13	0.24	6.05***
Age	0.09	0.04	0.09	2.27*	0.06	0.04	0.05	1.25	0.05	0.04	0.05	1.09
Sex × Age	–0.38	0.08	–0.18	−4.68***	–0.37	0.08	–0.18	−4.61***	–0.36	0.08	–0.17	−4.39***
Parent					2.48	1.23	0.09	2.01*	2.63	1.23	0.09	2.13*
Age × Parent									–0.18	0.09	–0.08	−1.97*
*R* ^2^		0.103				0.110				0.116		
*F* for change in *R*^2^		22.43				4.04				3.88		
*p*		< 0.001***				0.045*				0.049*		

*Sex (0 = male, 1 = female), Parent (0 = no, 1 = yes).*

*p < 0.05. **p < 0.01. ***p < 0.001.

When a similar hierarchical multiple linear regression was applied to cuteness rating data, adding parental status as a predictor variable did not increase *R*^2^-values significantly, *F*(1, 195) = 1.77, *p* = 0.185; *F*(1, 224) = 0.07, *p* = 0.792, for the mean rating of the 80 original faces and the mean rating of the 50 composite faces, respectively.

## Discussion

In the present study, a set of 50 facial images of Japanese 6-month-old infants was created, and their normative cuteness ratings were obtained in a Japanese population. For each face, two transformed versions were produced in which the cuteness level was augmented or reduced in reference to prototypical high- and low-cuteness faces. The two-alternative forced-choice task confirmed that the cuter face could be chosen from the pair better than at the chance level. Moreover, the sex and age of the respondent affected cuteness perception. Although the sex effect on the absolute values of cuteness rating was small and inconsistent, women gave more nuanced ratings to faces with different cuteness levels than did men in that women tended to give higher ratings to cuter faces and lower ratings to less cute faces than men did. Discrimination accuracy was lower for younger men than for older men and women of any age. Parents showed better discrimination performance than non-parents.

### Validity of the Japanese Cute Infant Face Dataset

The mean cuteness scores of the 10 cutest and 10 least cute faces that composed prototype faces were 4.39 and 3.15, respectively, on a 7-point scale. Although the numerical difference was small, they were similar to those in a previous study (4.6 and 3.3, averaging male and female babies; Sprengelmeyer et al., 2009). Cuteness scores of 50 composite faces ranged from 3.16 to 4.59 (*M* = 3.91, median = 3.93), which was around the midpoint (4), and no extreme values were obtained. However, reliable differences existed among the 50 images. There were 12 high-cuteness faces whose scores were significantly higher than 4 (i.e., 95% CI did not include and exceeded 4), 22 low-cuteness faces whose scores were significantly lower than 4, and 16 middle-cuteness faces in between. Therefore, infant faces with different levels of cuteness can be selected from the JCIF dataset.

Average faces were generally rated as cuter than individual composite faces. Even for the prototypical low-cuteness face, the score was 3.78 (95% CI [3.62, 3.93]), which was higher than the scores of 19 individual composite faces out of 50 (i.e., the 62nd percentile from the top). This is because average faces look more attractive (Langlois and Roggman, 1990; Langlois et al., 1994).

Choosing a cuter face from the pair was possible in all pairs of high- and low-cuteness versions of 50 composite faces (*M* = 88.0%, median = 90.4%). This expected result confirms that the cuter and less cute versions of infant faces in the JCIF dataset can be used as valid stimulus materials in cuteness research. Moreover, the accuracy of discriminating between manipulated faces and the mean cuteness ratings of base faces were negatively correlated (*r* = –0.42). That is, base faces with lower cuteness ratings were associated with higher discrimination accuracies. This means that shape manipulation was more effective for less cute faces than for cuter faces. A possible explanation is that originally cute infant faces may be less affected by cuteness manipulation because of a ceiling effect. In contrast, infant faces that were low in cuteness may have a greater chance of increasing the perceptual difference between the augmented and reduced cute faces. This pattern of results implies that people are more sensitive to a lack of cuteness cues than to the presence of the cues, which is described next.

### Asymmetrical Effects of Shape Manipulation on Cuteness Perception

Although the size of the physical difference was identical, the results showed that augmentation (+50%) and reduction (–50%) of cuteness had different effects on cuteness perception. The manipulation to reduce cuteness was more salient than the manipulation to augment cuteness. The data for average faces support this hypothesis. First, the effect of manipulation on cuteness ratings was larger for reduction (–50%) than for augmentation (+50%) (see Figure 2). Second, discrimination accuracy was higher for cuteness-reduced faces (–50%) than for cuteness-augmented faces (+50%) when they were presented in a pair with base faces (see Figure 3). The idea that people are more sensitive to less cute faces than to cute faces seems counterintuitive. According to Lorenz (1943) conception of Kindchenschema, responses to infant faces are evoked by the presence of certain physical features or cuteness cues, not by their absence. However, research on neural responses to infant faces suggests that, compared to cuter faces, less cute faces elicit larger responses at an early stage of perceptual processing. Using infant and adult faces that were manipulated in terms of cuteness (for infants) or attractiveness (for adults) by a transformation method similar to the present study, Hahn et al. (2016) reported that the amplitudes of early electrophysiological brain responses, N170 (120–200 ms) and P2 (200–250 ms), were larger for less aesthetic versions than for more aesthetic versions of both types of faces. This result is consistent with other studies showing that less attractive faces elicited larger neural responses than more attractive faces at an early stage of processing (Trujillo et al., 2014; Tagai et al., 2017). At later stages, however, cuter infant faces may induce larger reward-related processing than less cute infant faces (Glocker et al., 2009b; but see Bos et al., 2018; Endendijk et al., 2020, for null results).

In connection with this, the perception of infant faces and the perception of their cuteness are seemingly based on different mechanisms. Hahn et al. (2016) reported that infant faces elicited a larger N170 response than adult faces, although cuter infant faces elicited smaller N170 responses than less cute infant faces. Kringelbach et al. (2008) also reported that infant faces evoked neural responses at the orbitofrontal cortex as early as 130 ms, which were not observed for adult faces. Moreover, behavioral and electrophysiological studies have shown that infant faces attract more attention than adult faces at an early processing stage (Brosch et al., 2007, 2008; Holtfrerich et al., 2016). These results suggest that infant faces have processing advantage over adult faces, regardless of their cuteness level. Although often confused, the concept of cuteness is not identical to the concept of infantility or babyishness. Hildebrandt and Fitzgerald (1978) reported that the level of perceived cuteness affected the viewing duration of infant faces but did not modulate the strength of facial expressions measured by electromyogram, the latter of which uniformly increased with infant faces regardless of their cuteness level, and suggested two separable processes in infant face perception (see also Hildebrandt and Fitzgerald, 1983). Komori and Nittono (2013) showed that very infantile faces were rated as less cute and suggested that the evaluations of infantility and cuteness in infant faces were similar but dissociable. Moreover, there are types of cuteness that are not related to infantility or Kindchenschema (Nenkov and Scott, 2014; Nittono and Ihara, 2017). For these reasons, it is better to distinguish between overall reactions to infants in general and cuteness perception, which is the subject of the present study.

### Sex and Age Effects

The sex factor did not consistently affect the absolute level of cuteness ratings. The main effect of sex was observed only in the presurvey of the original faces. In contrast, the rater’s sex had different effects with different levels of cuteness. Compared to men, women tended to give lower scores to low-cuteness faces and higher scores to high-cuteness faces. This finding suggests that women are more sensitive to differences in cuteness. The difference value between the ratings of high-cuteness and low-cuteness faces has been used as an index of perceptual sensitivity (Hahn et al., 2015a,b). Taking this perspective into account, women’s lower ratings in the presurvey can be interpreted to reflect their higher sensitivity to cuteness perception. In the presurvey, women gave lower ratings than the midpoint, whereas men did not.

Conversely, the effect of age on cuteness ratings was inconsistent. No significant effects were obtained for the original and composite faces. For average faces, however, older people gave higher ratings. It is unclear why the age effect appeared only for average faces and how robust this finding is. Nevertheless, this does not mean perceptual sensitivity increased by age because no significant interaction effect was obtained between age and cuteness level (i.e., two prototypes or three manipulation levels). That is, the score differences between high- and low-cuteness faces did not change across age.

There was a clear interaction between sex and age on discrimination accuracy. While women’s performance was consistently good across all ages, men’s performance was lower at a younger age and increased with age. No sex difference was found in their 50s and 60s, although there was a possibility that the difference was masked by a ceiling effect. Because only the participants who answered correctly on a confirmation test of adult and baby faces, the poor performance of young men was unlikely to be due to an uncooperative and careless attitude. However, it was still possible that young men were less interested and engaged in discriminating between subtle differences in cuteness in infant faces than other subgroups. This result is consistent with the prediction that sex differences were greater at younger ages than at older ones (Sprengelmeyer et al., 2009). However, the previous and present results differ in two respects. First, they reported that old women (*n* = 12, 53–60 years old) and women after menopause (*n* = 10, *M* = 55.0 years old) showed as poor performance as young men (*n* = 24, 19–26 years old). Second, they reported that old men (*n* = 11, 53–60 years old) showed as poor performance as young men. According to their note, the average age at menopause was 51 years in Britain, which is about the same as that in Japan (median = 50.5 years) (Tamada and Iwasaki, 1995). Although the reason for the differences is unclear, the findings of the present study are reliable because a larger sample size and finer age segmentations were used than in the previous study. Moreover, the gradual reduction of sex differences from the 20s to 40s suggests that the obtained result was unlikely to be due to an artifact or noise in a specific age group. Poor performance in young men may be related to the level of male hormones. Research has shown that the level of testosterone in women modulated attention to infant faces (Holtfrerich et al., 2016) and that it modulated motivational behavior to see infant faces but did not modulate cuteness ratings (Hahn et al., 2015a). Further investigations are required to elucidate the relationship between perceptual sensitivity to cuteness and testosterone levels, especially in men. In contrast, the present study did not suggest any effect of female hormones because women’s performance did not show an age effect and remained high until their 60s.

Research has shown sex/gender and age differences in interest in and attitude toward infants and cuteness (Robinson et al., 1979; Berman, 1980; Maestripieri and Pelka, 2002; Nishiyama et al., 2015). A recent cross-cultural study showed that young women had more positive attitudes toward cute things (not restricted to infants) than men, and this tendency decreased with age, which was similar in Japan, the United States, and Israel (Nittono et al., 2021). However, the present study did not show any evidence for young women having superior performance than other groups in cuteness perception.

### Effects of Parental Status

Even after controlling for the effects of sex and age statistically, parental status was associated with a higher sensitivity to cuteness in infant faces. The effect of parenthood was reduced with age, partly because of a ceiling effect. On average, those who had a child or children showed 2.63% (95% CI [0.21, 5.05]) greater accuracy than those who did not (see Model 3 in Table 2). Although this was smaller than the sex effect (6.81%, 95% CI [4.60, 9.03]), it was statistically significant. Additionally, there was no interaction effect of parental status with sex. These results are consistent with a previous finding that parental status, independently of sex/gender, was associated with more positive affective responses toward infants than adults (Lehmann et al., 2013). Parental status has been shown to be associated with lower levels of testosterone in both men and women (Kuzawa et al., 2010; Gettler et al., 2011). If perceptual sensitivity is related to testosterone level, this hormonal change may explain the improved performance. Although Hahn et al. (2015a) reported that the mean cuteness rating differences between the 50% and –50% versions of infant faces did not covary with salivary testosterone levels in women, different results may be obtained using discrimination accuracy in a two-alternative forced-choice task, which is shown to be a more sensitive measure than ratings in the present study.

Because this study was a correlational study, no causal relationship can be derived. It is also possible that those who have a better perceptual ability with cuteness are more interested in children and make a choice to have children. In a future study, intra-individual changes in sensitivity before and after becoming a parent would be worth investigating to elucidate the process.

## Limitations and Future Directions

This study has several limitations. First, the original facial images were not taken under identical conditions. Although their qualities were carefully controlled, they might not be optimal. Nevertheless, because normative ratings and discrimination performance were obtained from these images, the dataset itself is valid. Second, the data were obtained from an online survey. It was impossible to precisely control for the participants’ environments or the size of the images presented. However, as each participant responded to all the stimuli in a constant condition, relative differences among stimuli are assumed to be reliable. Third, only two types of transformations (+50% and –50%) were applied. This combination has often been used in previous studies (Hahn et al., 2013, 2015a,b,^2016^). Because the discrimination accuracy was high (*M* = 88.0%, median = 90.4%), they are suitable for studies that require obvious differences between conditions. However, they may not be suitable for studies that require more subtle differences in cuteness levels. To measure perceptual sensitivity, pairs with smaller differences (±25% or ±12.5%) would be preferable (Sprengelmeyer et al., 2009; Lobmaier et al., 2010, 2015). New images can be easily created in Psychomorph software using the templates of prototypical and base faces of this study, which are publicly available. Fourth, the normative ratings were obtained from a Japanese population. This method is natural because it is ecologically valid that both adult raters and infant targets belong to the same ethnic population. The Japanese prototypical faces created here appear to share similar morphological characteristics with the Caucasian prototypical faces produced on the basis of ratings of Westerners (e.g., Sprengelmeyer et al., 2009). Previous studies have reported that cuteness ratings did not vary between ingroup and outgroup babies (Spencer et al., 2018) and that preference for cuter babies did not depend on their ethnicity (Golle et al., 2015). However, the absolute levels of cuteness ratings can differ in different populations (Volk, 2009). Fifth, the present study dealt only with perceptual sensitivity to cuteness. Sex and age may affect motivational and behavioral outcomes differently (e.g., Parsons et al., 2011; Hahn et al., 2015b). Such studies could be conducted using the current dataset. Finally, normative ratings other than cuteness are not obtained. Although cuteness ratings are sufficient for the original purpose, ratings on other dimensions such as typicality and distinctiveness might be helpful to enhance the applicable scope of this dataset.

## Conclusion

The JCIF dataset developed in the present study can be a useful tool for future research in this field. Despite increasing interest in cuteness research, there has been no specialized dataset of infant faces created, partly because of the portrait rights problem. As a dataset of child facial images, the Child Affective Facial Expression (CAFE) set is available (LoBue and Thrasher, 2015). However, the age of children included in that dataset (2–8 years old) is older than those usually used in infant cuteness research. Because the JCIF dataset contains 6-month-old infant faces with a neutral expression, it is more suitable for perceptual and cognitive experiments using infant faces.

## Data Availability Statement

The datasets presented in this study can be found in online repositories. The names of the repository/repositories and accession number(s) can be found below: https://osf.io/pseym/.

## Ethics Statement

The studies involving human participants were reviewed and approved by the Behavioral Research Ethics Committee of the Osaka University School of Human Sciences (HB30-033). The patients/participants provided their written informed consent to participate in this study.

## Author Contributions

HN, AO, and MK contributed to conception and designed of the study. HN and AO collected stimulus materials, conducted online surveys, and performed the statistical analysis. AO processed facial images. HN wrote the draft of the manuscript and made figures and tables. All authors contributed to manuscript revision, read, and approved the submitted version.

## Conflict of Interest

The authors declare that the research was conducted in the absence of any commercial or financial relationships that could be construed as a potential conflict of interest.

## Publisher’s Note

All claims expressed in this article are solely those of the authors and do not necessarily represent those of their affiliated organizations, or those of the publisher, the editors and the reviewers. Any product that may be evaluated in this article, or claim that may be made by its manufacturer, is not guaranteed or endorsed by the publisher.

## Footnotes

1
http://users.aber.ac.uk/bpt/jpsychomorph/

2
https://osf.io/pseym/
