# Supplementary material for: Creation and Validation of the Japanese Cute Infant Face (JCIF) Dataset
Source: Front Psychol. 2022 Feb 18;13:819428. doi: 10.3389/fpsyg.2022.819428 (PMC8895142; doi:10.3389/fpsyg.2022.819428)
Supplement: Supplementary file 1 [file Data_Sheet_1.pdf]

Supplementary Material

Supplementary Table 1. The numbers of parent and non-parent respondents in each subgroup

	Female			Male			Total		
Parent	Yes	No	Sum	Yes	No	Sum	Yes	No	Sum
Presurvey									
20s	2	18	20	4	16	20	6	34	40
30s	11	9	20	3	17	20	14	26	40
40s	13	7	20	8	12	20	21	19	40
50s	14	6	20	11	9	20	25	15	40
60s	16	4	20	16	4	20	32	8	40
Sum	56	44	100	42	58	100	98	102	200
Rating									
20s	6	16	22	0	21	21	6	37	43
30s	11	12	23	13	13	26	24	25	49
40s	11	9	20	10	14	24	21	23	44
50s	17	6	23	16	6	22	33	12	45
60s	20	6	26	15	7	22	35	13	48
Sum	65	49	114	54	61	115	119	110	229
Discrimination									
20s	13	47	60	4	48	52	17	95	112
30s	29	30	59	14	44	58	43	74	117
40s	36	24	60	31	30	61	67	54	121
50s	37	22	59	33	24	57	70	46	116
60s	46	12	58	43	20	63	89	32	121
Sum	161	135	296	125	166	291	286	301	587

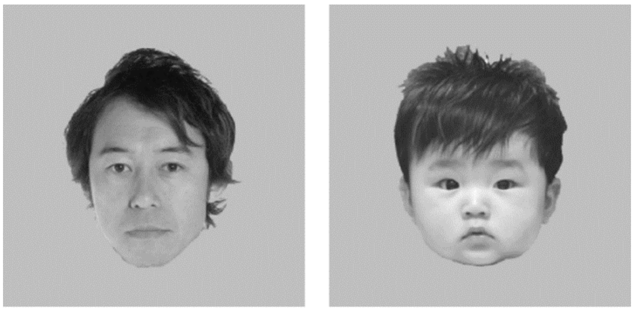

Supplementary Figure 1. A stimulus example of the adult vs. infant face choice task in which the participants were asked to choose the infant face from the pair. This task was to confirm that the participants performed the task conscientiously by watching the images carefully. The adult face depicted here is a substitute image that is similar to the original ones, because the original adult images were copyrighted. The facial images here were taken from a royalty-free image website (https://www.photo-ac.com/).

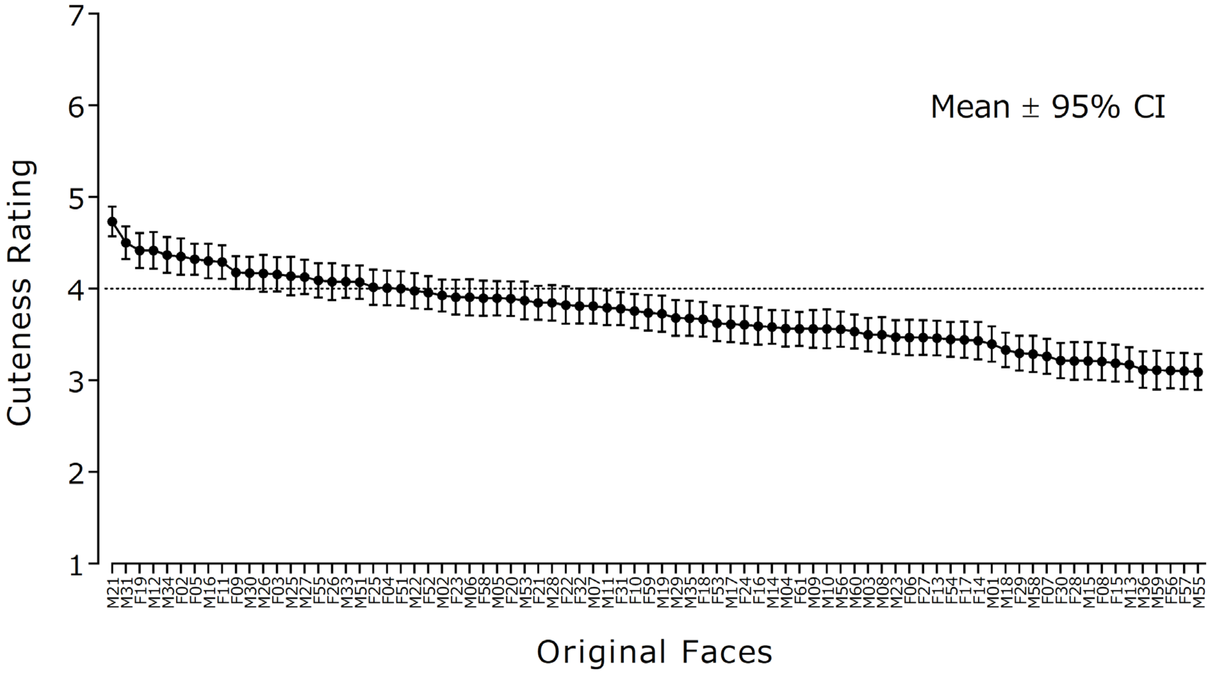

Supplementary Figure 2. Mean cuteness rating scores for 80 original faces ($N = 200$). Error bars indicate 95% confidence intervals.

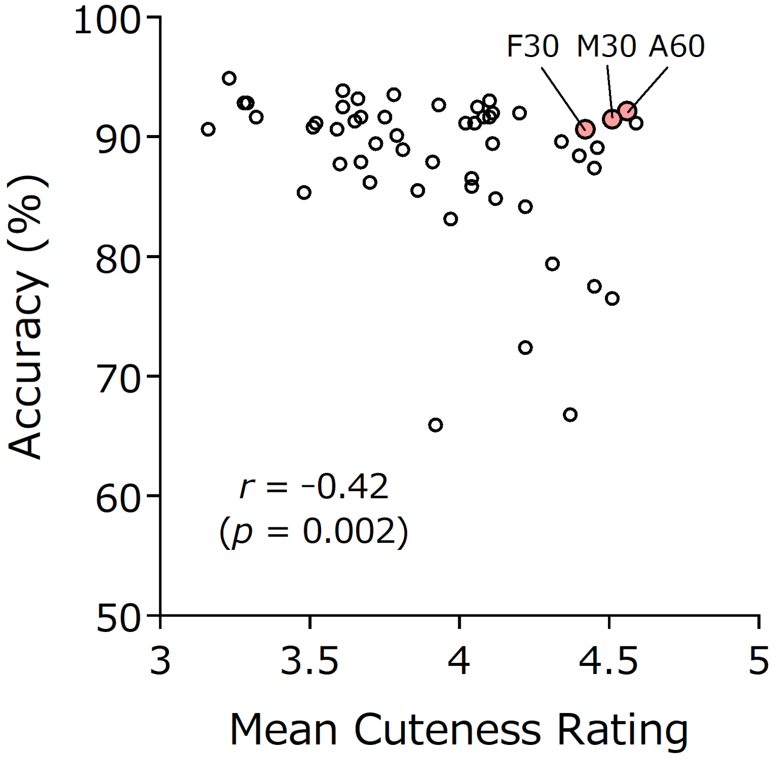

Supplementary Figure 3. The relationship between mean cuteness ratings and discrimination accuracies of 50 composite faces.

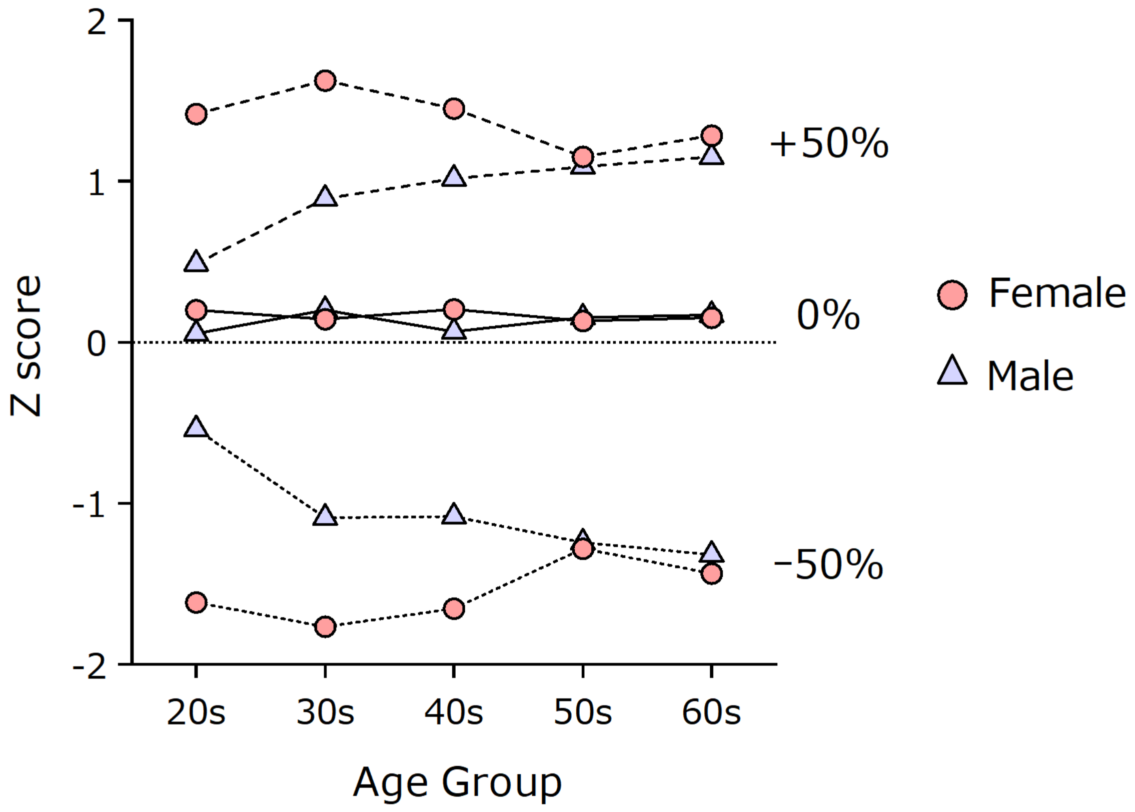

Supplementary Figure 4. Scale values calculated by Thurston's paired comparison method. The higher values indicate the higher cuteness estimations. Note that average faces (0%) were associated with more positive values than zero, indicating that average faces were not neutral but, rather, were perceived to be slightly cute.

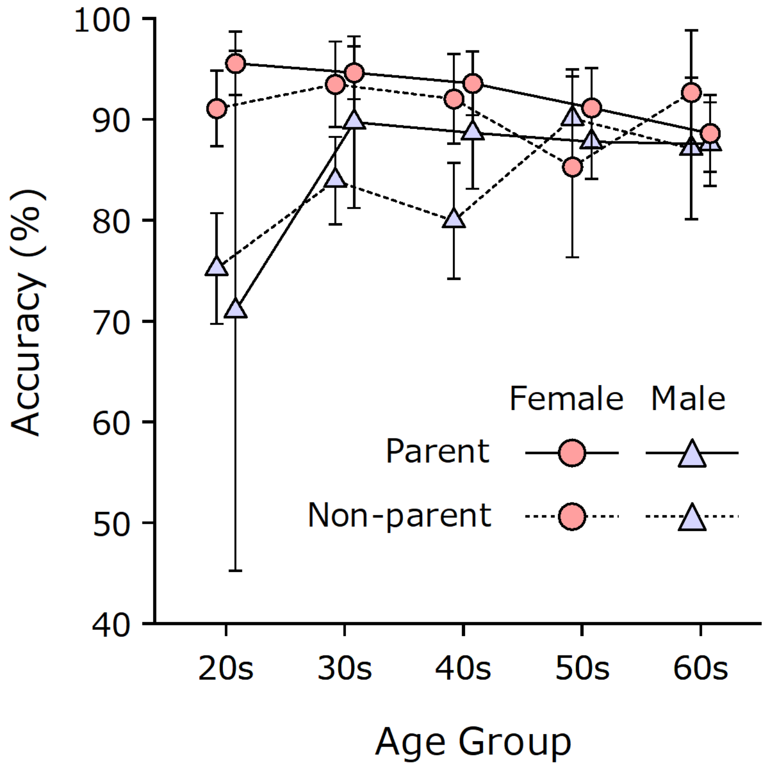

Supplementary Figure 5. The relationship between parental status and cuteness discrimination accuracy. Error bars indicate 95% confidence intervals. Note that parent ratio differed in each subgroup (see **Supplementary Table 1**).
